# Mediterranean Diet, Screen-Time-Based Sedentary Behavior and Their Interaction Effect on Adiposity in European Adolescents: The HELENA Study

**DOI:** 10.3390/nu13020474

**Published:** 2021-01-30

**Authors:** Miguel Seral-Cortes, Sergio Sabroso-Lasa, Alexandro Bailo-Aysa, Marcela Gonzalez-Gross, Dénes Molnár, Laura Censi, Cristina Molina-Hidalgo, Frederic Gottrand, Stefaan De Henauw, Yannis Manios, Christina Mavrogianni, Kurt Widhalm, Anthony Kafatos, Jean Dallongeville, Luis A. Moreno, Luis Mariano Esteban, Idoia Labayen, Pilar De Miguel-Etayo, on behalf of the HELENA Study Group

**Affiliations:** 1 Growth, Exercise, NUtrition and Development (GENUD) Research Group, Faculty of Health Sciences, Instituto Agroalimentario de Aragón (IA2), Instituto de Investigación Sanitaria Aragón (IIS Aragón), Universidad de Zaragoza, 50009 Zaragoza, Spain; lmoreno@unizar.es (L.A.M.); pilardm@unizar.es (P.D.M.-E.); 2 Genetic and Molecular Epidemiology Group (GMEG), Spanish National Cancer Research Centre (CNIO), 28029 Madrid, Spain; ssabroso@cnio.es; 3 Department of Physiatry and Nursing, Faculty of Health Sciences, Universidad de Zaragoza, 50009 Zaragoza, Spain; abailoaysa@gmail.com; 4 CIBER Fisiopatología de la Obesidad y Nutrición (CIBERobn), Instituto de Salud Carlos III, 28029 Madrid, Spain; marcela.gonzalez.gross@upm.es; 5 ImFine Research Group, Department of Health and Human Performance, Facultad de Ciencias de la Actividad Física y del Deporte-INEF, Universidad Politécnica de Madrid, 28040 Madrid, Spain; 6 Institute of Nutritional and Food Sciences, Nutritional Physiology, University of Bonn, 53113 Bonn, Germany; 7 Department of Pediatrics, Medical School, University of Pécs, 7623 Pécs, Hungary; molnar.denes@pte.hu; 8 Department of Applied Science of Nutrition, Council for Agricultural Research and Economics, Research Center for Food and Nutrition, 00198 Rome, Italy; laura.censi@crea.gov.it; 9 EFFECTS 262 Department of Medical Physiology, School of Medicine, University of Granada, 18071 Granada, Spain; criismh@correo.ugr.es; 10 CHU Lille, Inserm U1286 INFINITE, University of Lille, F-59000 Lille, France; Frederic.GOTTRAND@CHRU-LILLE.FR; 11 Department of Public Health and Primary Care, Faculty of Medicine and Health Sciences, Ghent University, 9000 Ghent, Belgium; Stefaan.DeHenauw@UGent.be; 12 Department of Nutrition and Dietetics, School of Health Science & Education, Harokopio University, 176 71 Athens, Greece; manios@hua.gr (Y.M.); cmavrog@hua.gr (C.M.); 13 Division of Clinical Nutrition and Prevention, Department of Paediatrics, Medical University of Vienna, Austria and Austrian Academic Institute for Clinical Nutrition, 1090 Vienna, Austria; kurt.widhalm@meduniwien.ac.at; 14 Faculty of Medicine, University of Crete, 715 00 Crete, Greece; kafatos@med.uoc.gr; 15 Department of Epidemiology Public Health, Institut Pasteur de Lille, 59800 Lille, France; jean.dallongeville@pasteur-lille.fr; 16 Escuela Politécnica de La Almunia, Universidad de Zaragoza, 50100 Zaragoza, Spain; lmeste@unizar.es; 17 Department of Health Sciences, Public University of Navarra, 31006 Pamplona, Spain; idoia.labayen@unavarra.es

**Keywords:** Mediterranean diet, sedentary time, adiposity, adolescents, gender and HELENA

## Abstract

Childhood obesity is a worldwide epidemic. Mediterranean diet (MD) is inversely associated with childhood obesity, but the interaction with other environmental factors, such screen time, might influence the health benefits of a high MD adherence in adolescents. The aim of the present study was to assess whether an association between MD and screen time exists in European adolescents. Moreover, we also explored whether sedentary time has a modulatory effect on the association between MD and adiposity. Adherence to the MD (24 h recalls), screen time (questionnaire), pubertal development, body mass index (BMI), fat mass index (FMI) and waist circumference (WC) were evaluated in 2053 adolescents (54.7% females), aged 12.5–17.5 years. In females, MD adherence was associated with lower BMI and FMI only when they were exposed to less than 338 min/day of screen time (81.8% of females); MD adherence was also associated with lower WC only when females were exposed to less than 143 min/day of screen time (31.5% of females). No significant MD-screen time interaction was observed in males. In conclusion, screen-time-based sedentary behaviours had a modulatory effect in the association between MD adherence and adiposity in European female adolescents.

## 1. Introduction

Childhood overweight and obesity’s prevalence has been rising worldwide in recent years [1]. Recent studies showed that overall, European children continue to struggle with high prevalence of obesity despite the effort in terms of prevention programs in previous years [2]. Metabolic syndrome and type 2 Diabetes are more likely to occur in adulthood in those children and adolescents where obesity is gradually establishing [3]. The Mediterranean dietary pattern is inversely associated with childhood obesity [4]. In fact, a high Mediterranean diet (MD) adherence from an early age is related to a lower risk of overweight and obesity development in childhood [5]. However, the interaction with other environmental factors, such as screen time, might influence the health benefits of a high MD adherence in adolescents. Sedentary behaviours, such screen time and physical inactivity were found to increase the risk of overweight and obesity in European adolescents [6,7]. Current recommendations suggest limiting recreational screen time to less than 2 h per day [8]. However, previous studies have shown that more than half of all children exceed screen time recommendations [9,10,11]. Additionally, increasing sedentary time is associated with unhealthy dietary patterns in European adolescents [12]. Furthermore, there is a growing evidence of transition from the traditional MD pattern into consumption of energy-dense foods, such as in the Western diets, especially in Mediterranean countries [13,14,15,16].

Previous data showed an inverse association between MD adherence and sedentary time, including screen time [17,18]. However, to our knowledge, a potential interaction effect between MD and screen time on adiposity remains unknown. Therefore, the aim of the present study is to assess whether an association between MD and sedentary time exists in European adolescents in the Healthy Lifestyle in Europe by Nutrition in Adolescence (HELENA) study. Moreover, we intend to explore whether sedentary time has a modulatory effect on the association between MD and adiposity markers. We hypothesize that high levels of screen time may attenuate the protective effect of MD adherence on adiposity parameters.

## 2. Materials and Methods

### 2.1. Study Design and Population

**Figure 1:**
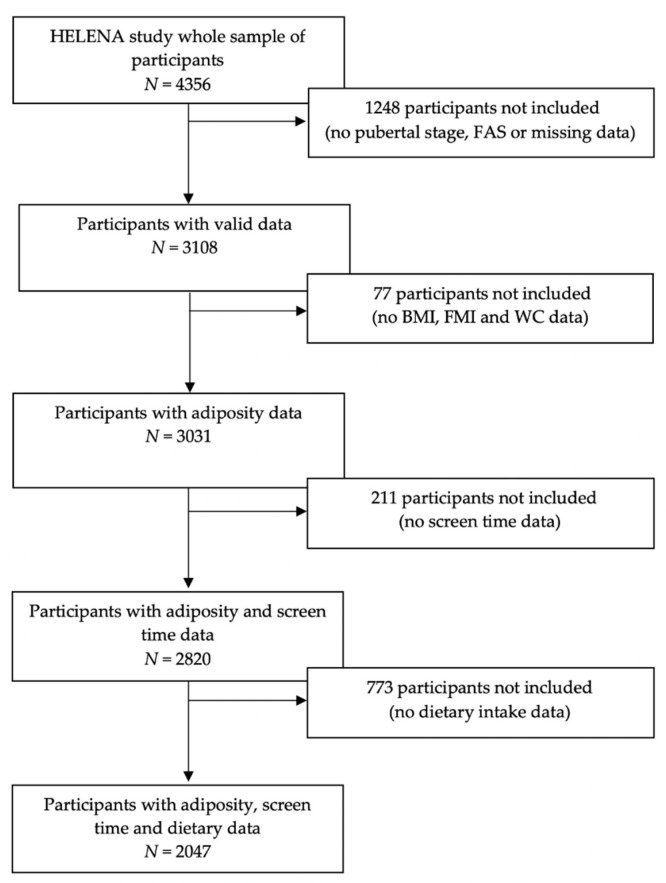
Flow chart of the sample selection process. Abbreviations: FAS, family affluence scale; BMI, body mass index; FMI, fat mass index and WC, waist circumference.

HELENA is a multicentre and cross-sectional study. Description of the study sampling and recruitment, standardization and harmonization methodology, data collection, analysis strategies and quality control procedures was published elsewhere [19,20]. The HELENA study was designed to obtain reliable and comparable information on adolescents’ nutritional, environmental and health-related influences to prevent risk factors for present and future nutrition-related chronic diseases [21]. Each one of the participating countries involved in the HELENA study approved the protocol by the local Research Ethics Committees and followed the ethical guidelines of the Declaration of Helsinki 1964 (revision of 2000), the Good Clinical Practice and the legislation about clinical research in humans [22]. A written consent was provided to the parents or guardians of all individuals participating in the study, which was read and signed. The present study comprises 2047 adolescents (54.7% females), aged 12.5–17.5 years, with valid and specific data on adherence to MD, screen time and adiposity. Description of the selection process is shown in a flow chart (Figure 1).

### 2.2. Physical Examination and Adiposity Measurements

Anthropometric measurements were strictly controlled and performed following standard protocols [23]. Body height was measured barefoot with a telescopic stadiometer (SECA 225) to the nearest 0.1 cm. Body weight was measured in underwear and with no footwear to the nearest 0.1 kg with an electronic scale (SECA 861, Hamburg, Germany). Height and weight were measured in triplicate. Body mass index (BMI) was calculated by dividing weight (kg) by the square of height (m) [24]. Waist circumference (WC) measurements were performed with a non-elastic tape (SECA 200) to the nearest 0.1 cm at the mid-point between the lowest rib and the iliac crest. Subscapular and tricipital skinfold thicknesses were measured in triplicate. In order to assess the contribution of fat mass relative to body size, the body fat percentage was calculated using the Slaughter’s equation [25], and then, FMI was calculated as body fat in relation to height squared [FM (kg)/height (m^2^)] [26]. Pubertal status was evaluated during a medical examination by a physician/paediatrician following the methodology described by Tanner and Whitehouse [27]. Pubertal status was categorized as Tanner stages from no sexual maturation (stage I) to complete sexual maturation (stage V).

### 2.3. Dietary Intake and Mediterranean Diet Score (MDS) Assessment

The HELENA Dietary Assessment Tool (HELENA-DIAT) is a self-administered computerized 24 h dietary recall used to collect all the adolescents’ dietary intake [28,29]. This tool was first validated in Flemish adolescents [29] and then adapted to be implemented in the participating centres of each country [30]. Participants provided twice dietary information through the HELENA-DIAT on 2 non-consecutive days within a space of 2 weeks. Previous authors considered this method as an useful procedure to evaluate the dietary intake in European children and adolescents [31]. The multiple source method (MSM) allowed us to calculate usual dietary intake of each individual, which enables the possibility to correct the dietary information for between and within individuals´ variability [32].

A Mediterranean diet score (MDS) was computed from the sum of 9 single subcomponents that were described elsewhere [33]. In short, vegetables, fruits and nuts, cereals, legumes, fish, dairy products (recommended during growth and development periods [34]) and unsaturated to saturated fat ratio were considered healthy food subgroups of MD, whereas meat products (including processed meat) and alcohol consumption were classified as unhealthy factors. Therefore, a participant consuming a healthy MD-associated food group was designated with 1 point, whereas the unhealthy food subgroups contributed with −1 points. A Mediterranean diet score (MDS), showing the degree of adhesion to the MD for each individual, was developed using a 0–9 point scale, with low values (0–4) indicating poor adherence and high values (5–9) greater adherence, respectively [35,36]. Supplementary Table S1 shows the median intake in g/day by sex of each subgroup from the MDS and the adherence levels to the MD.

### 2.4. Screen-Time-Based Sedentary Behavior Assessment

In order to report the habitual time devoted to screen time among adolescents, a validated self-reported screen-time-based sedentary behaviours questionnaire was used [37]. The time spent in TV viewing, computer games, video games and internet for non-study reasons during both week and weekend days was collected in categories in a scale ranging from 0–240 min per day. The daily mean time for each category was obtained and the final time was calculated summing weekdays and weekend days, obtaining the total screen time in minutes per day (min/day). Lastly, a total sedentary time value was obtained by summing up the time reported in each category. The weighted Cohen’s κ-coefficients were used to assess the test–retest reliability of the screen-time-based questionnaire used in the HELENA study. The most common values observed were moderate, substantial or almost perfect agreement (>0.7). Exceptionally, internet for study reasons showed 0.46 in weekdays and 0.33 in weekends, respectively [38]. Furthermore, a sensitivity analysis was carried out in order to discard potential disparities in the interaction models due to those outlier individuals considered (or not) in the screen time variable.

### 2.5. Socioeconomic Status

The family affluence scale (FAS) is an indicator of material affluence, which ranges from 0 (lowest) to 8 (highest) and further recategorized in low (0–2), medium (3–5) and high (6–8) levels [39]. The scale considers parameters such as car ownership, having an own bedroom, internet availability and computer ownership. This information was assessed through a questionnaire, and it was used as a predictor of the adolescents’ health outcomes [40].

### 2.6. Statistical Analysis

The normality of the variables was assessed with the Shapiro–Wilk non-parametric test. Not all variables followed a normal distribution, so the descriptive sex-specific characteristics are shown as median and interquartile range (IQR) for continuous variables, while categorical variables are shown as absolute and relative frequencies. Moreover, Pearson’s chi-square statistical test was used to obtain comparative sex-related differences for categorical variables; the Mann–Whitney–Wilcoxon test was performed for continuous variables. In order to observe the association between MD and screen time, sex-specific multiple linear regression models were performed. First, a raw simple linear regression model was constructed to observe associations between MD and screen time. Then, an initial multiple linear regression model was performed considering energy intake, socioeconomic status and Tanner stage as confounders. A step-by-step algorithm was applied to select the significant variables in a multivariate model to shortlist the independent variables significantly associated with the adiposity parameters in the final model. Furthermore, a new multiple regression analysis was created to assess the association between adiposity parameters and MD, adding the screen time interaction effect, the MD effect alone and the abovementioned confounders. Finally, as we observed extreme values for screen time in the highest end of the distribution, we performed a sensitivity analysis, excluding the outliers in the top end of the distribution. Level of significance was set at *p* < 0.05. RStudio Version 1.2.5001 (RStudio Team (2015). RStudio: Integrated Development for R. RStudio, Inc., Boston, MA, USA, URL http://www.rstudio.com/) was used to perform all statistical analyses.

## 3. Results

### 3.1. Descriptive Characteristics of the Study Sample

Table 1 shows the main characteristics of the HELENA participants included in the present study. Summarizing, males had higher weight, height and WC (*p* ≤ 0.001), and lower FMI than females (all *p* < 0.001). Moreover, males were more exposed to screen time (*p* ≤ 0.001) and had higher energy intake (*p* ≤ 0.001) than females, although females were in more advanced pubertal stages than males (*p* ≤ 0.001). Finally, there were no significant differences regarding FAS and MDS.

**Figure 2:**
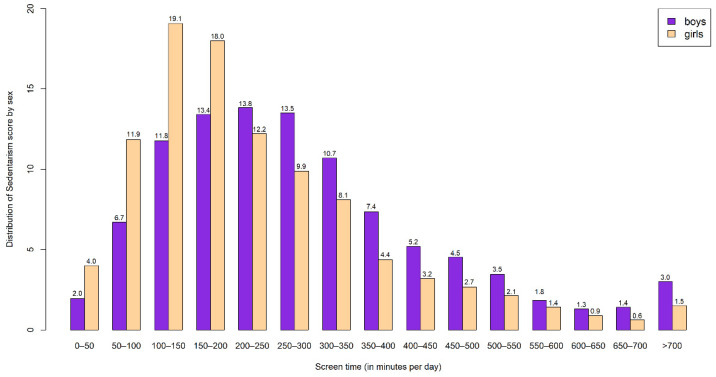
Distribution of screen time (% displayed) among HELENA participants by sex.

The screen time distribution (min/day) between HELENA participants is shown in Figure 2.

### 3.2. Association between MD Adherence and Screen-Time-Based Sedentary Behaviors

The associations between MD and screen time are shown in Table 2. The univariate model (Model I) showed that MD and screen time were inversely associated in both males and females (*p* < 0.001). These relationships were maintained in the initial multivariate model (*p* < 0.001) and after adjusting by confounders (Model II; *p* < 0.001).

### 3.3. Interaction between MD Adherence and Screen-Time-Based Sedentary Behaviors on Adiposity

The interaction effects between MD and screen time on adiposity parameters by sex group are displayed in Table 3. In males, the screen-time–MD interaction was not significantly associated to any adiposity index. However, in females, there were significant interaction effects between the screen time and MD on BMI (*p* < 0.05), WC (*p* < 0.01) and FMI (*p* < 0.05) (Table 3).

**Figure 3:**
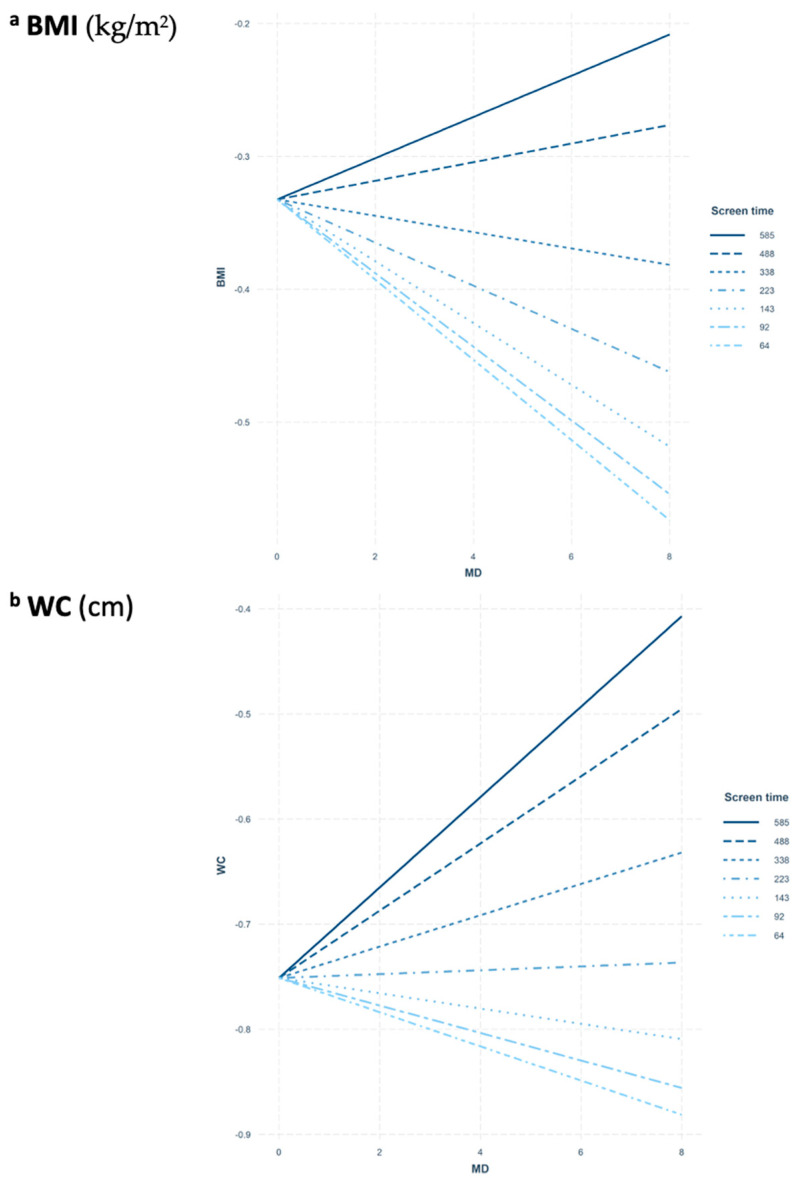

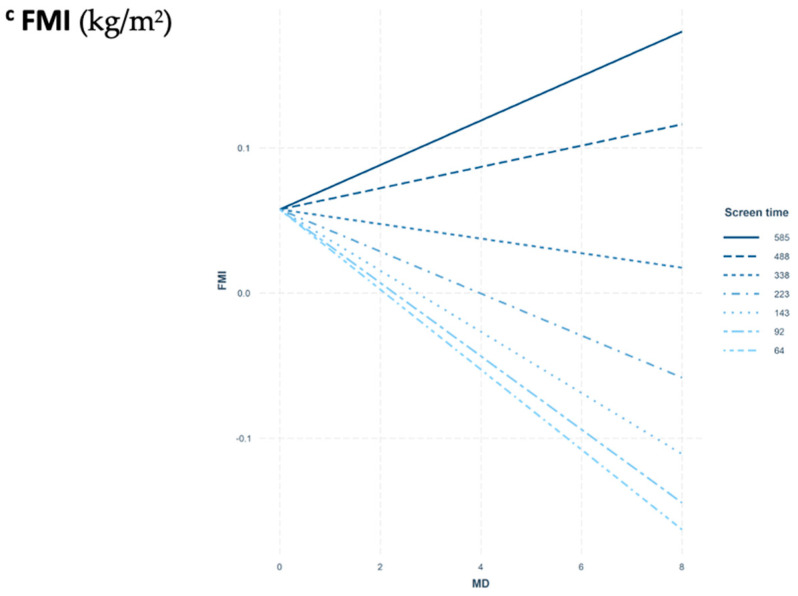
Matrix panel of interaction models on (**a**) BMI (body mass index), (**b**) WC (waist circumference), (**c**) FMI (fat mass index); and MD (Mediterranean diet) according to screen time modulation in females. In order to design the representation of the distribution in HELENA adolescents, different lines were traced as reference points to observe the slope of the studied population according to the sedentary time. A positive gradient represents the MD acting as risk factor, while a negative gradient shows the MD acting as protective factor.

In order to interpret the modulation effect of screen time on the relationship between MD and adiposity indices in females, a set of figures is displayed in a matrix panel related to each adiposity index (Figure 3). A number of lines were drawn to represent the MD and adiposity variables modulated by the distribution of the screen time. Most participants were located in lower and central parts of the distribution, corresponding to 50–350 min/day (74.5% of the total population); considering this distribution, the impact of outliers was carefully considered. Despite the observed screen time habits, those individuals represented by a negative slope could benefit from the protective role of MD in relation to adiposity indices when the MD adherence is high. Therefore, a high MD adherence was associated with lower BMI only in those females being exposed to screen time less than 338 min/day (81.8% of the total females). Moreover, high MD adherence was associated with lower WC only in those females being exposed to screen time less than 143 min/day (31.5% of total females). Finally, a high MD adherence was associated with lower FMI only in those females being exposed to screen time less than 338 min/day (81.8% of total females).

### 3.4. Sensitivity Analysis for Screen-Time-Based Sedentary Behaviors Plausible Data

For the significant interaction models (females), sensitivity analysis was performed considering screen time outliers (highest value: 856 min/day) vs. interaction models not considering outliers (highest value: 632 min/day) on adiposity indices. Minimal differences were observed in the two interaction models of each adiposity parameter (β < 0.001 vs. β < 0.001 in BMI, WC and FMI).

## 4. Discussion

The main findings of the present study are the observed inverse association between MD adherence and screen time, and the joint interaction effect between both factors on adiposity in European female adolescents. Thus, the benefits associated with a high MD adherence were only observed in those females with lower screen time. On the other hand, no MD–screen-time interaction effect was observed in males.

In line with our findings, previous studies reported an inverse association between MD adherence and sedentary time among youth. In a population of Mediterranean European adolescents, self-reported inactivity [41] was inversely related to MD adherence in both sex groups [42]. In non-Mediterranean European adolescents, a low MD adherence was associated with higher sedentary time, although their sedentary time assessment only considered sitting time during weekdays [43].

To our knowledge, no other studies have examined screen time as modulatory factor in the MD effect on adiposity indices. However, a combined effect of different lifestyle patterns in cluster studies assessing their relationship to adiposity has been reported in similar age populations [44,45,46,47]. Regarding the combined effect of unhealthy habits, in European children, a cluster including high sedentary activities (including screen time), low PA, sweet beverages and low fruits and vegetables intake was associated with high BMI and WC [44]. Similar results were found in non-European children, where an unhealthy cluster of TV viewing with energy dense foods was associated to high BMI [45]. Although we are considering cross-sectional studies, similar associations were also obtained in longitudinal studies in European [46] and non-European children [45]. In the same way, associations between clusters of healthy lifestyle patterns and low levels on adiposity indices were observed in Spanish youth [47].

Cluster analysis could be useful to evaluate the combined effect of environmental and behavioural factors on overweight and obesity among children and adolescents [47,48]. However, we found no studies that have considered the interaction effect between the factors included. Yet, a recent study conducted in a large sample of adolescents showed that screen time was associated with increased risk of overweight/obesity, regardless of being combined with other healthy or unhealthy behaviours [49]. This finding suggests that high levels of screen time might mask the beneficial effects of a healthy diet, such as the MD, on decreasing the obesity risk. Previous studies in children have reported similar co-existing effects among behaviours [50,51], which supports the findings of our study; low levels of PA might also counteract the beneficial effect of a healthy dietary pattern [52].

It remains challenging to find the potential mechanisms responsible for gender disparities in the obtained results. As in other studies in European adolescents [53], we found that the time spent in screen time activities was higher in males than in females. However, conflicting data were found in this regard [43]. Screen time could be an indicator of gender variability, but further investigation is required to obtain comparable data to explain gender differences in the present analysis. Another hypothesis that might explain the gender differences could be the poorer sleep duration and thus increased adiposity in females than males, previously observed in other similar age cohorts [54]. Short sleep duration was already observed to be associated with increased adiposity biomarkers in HELENA adolescents, particularly in females [55], and with lower dietary quality [56]. Further gender differences were also observed in a cluster study comprising PA, sedentary behaviours and sleep duration according to endocrine, metabolic and immunological biomarkers, which suggests new pathways to focus our future research [57].

In the current study, a sensitivity analysis was carried out in order to detect potential differences in the results obtained considering vs. not considering outliers in the number of hours of screen time. There were no significant differences between the two proposed models assuming vs. not assuming those extreme values; therefore, the final results were not affected by those specific differences in screen time.

In addition, a different effect was observed in the studied adiposity indices depending on the amount of screen time, requiring less screen time in those females to obtain lower WC levels than BMI and FMI, while higher levels were observed in those females with higher screen time. Similar findings were observed in terms of BMI and WC differences in previous studies in adolescents [6].

The current study presents some limitations. Due to the cross-sectional nature of the HELENA study, cause–effect relationships cannot be concluded. We assume the challenging task of collecting dietary data through the self-administered computerized 24 h dietary recall in adolescents; therefore, reliability of answer depends completely on the responders´ interpretation. Although the two non-consecutive days assessment of dietary habits might not consider certain periods of time such as weekends or holidays, this method has previously shown good validity and accuracy in similar populations [58]. An identical situation occurs for the self-reported questionnaire to obtain measures of screen-time-based sedentary behaviours, where we also acknowledge that screen time might vary between weekdays and weekend days; yet, this method was adapted and validated to be used in adolescents [37]. Additionally, other screen time devices such as tablets, phones and consoles are becoming more dominant over the traditional TV viewing routines; so, future studies should consider them in their screen-time-based sedentary behaviours assessment. However, some strengths should also be acknowledged. Firstly, it is important to highlight the high standard of implementation in terms of methodology and design of the HELENA study [19,21], as well as the large number of sedentary behaviours considered to estimate screen time in the present cohort of participants. Furthermore, datasets with a large number of adolescents diversely recruited across Europe have been provided as a result of the multicentric nature of the HELENA study. This fact allows researchers to focus their analysis on an age range which is rarely studied, the adolescence period, where early detection of adverse sedentary and dietary habits might influence their health status in adulthood. Finally, little has been found in the literature considering the same interaction approach, where screen time plays an important role in the association of MD and adiposity among adolescents.

As we have observed, the negative association between screen time and MD adherence on adiposity indices, joint public health implementation programs oriented to both, avoiding excessive screen time and promoting the MD dietary pattern for primary prevention of major chronic diseases, should be considered in the near future. In previous childhood obesity prevention programs, the greatest changes were observed in sedentary time [59]. At the same time, previous literature has shown that the most effective interventions to prevent childhood obesity are the ones combining diet and PA/sedentarism [60]. The evidence of co-existing effects might help to understand how screen time and dietary behaviours should be approached simultaneously in prevention programs.

## 5. Conclusions

Screen time and MD adherence are inversely associated in European adolescents. Moreover, in females, screen time had a modulatory effect in the association between MD adherence and adiposity in European adolescents. These findings support the idea of applying more personalized public health recommendations in reducing screen time to benefit from the MD, in order to decrease the adiposity levels among youth.

## Figures and Tables

**Table 1:** Demographics and behavioural characteristics of the Healthy Lifestyle in Europe by Nutrition in Adolescence (HELENA) participants displayed by sex.

	Total	Male	Female	*p*
	*n* = 2047	*n* = 925	*n* = 1122	
**Age** (years)	14.7 (13.7–15.7)	14.8 (13.7–15.7)	14.7 (13.7–15.7)	0.590
**Height** (cm)	165.9 (159.3–172.0)	170.1 (163.9–177.1)	162.4 (157.7–167.0)	**<0.001**
**Weight** (kg)	58.4 (50.3–64.3)	61.5 (52.3–68.9)	55.8 (49.0–61.2)	**<0.001**
**BMI** (kg/m^2^)	21.1 (18.7–22.8)	21.1 (18.6–22.8)	21.1 (18.8–22.8)	0.282
**WC** (cm)	71.8 (66.2–75.8)	73.8 (67.8–78.3)	70.2 (65.0–74.4)	**<0.001**
**FMI** (kg/m^2^)	5.15 (3.1–6.3)	4.5 (2.4–5.3)	5.7 (4.0–6.8)	**<0.001**
**Pubertal stage** [*n* (%)]				**<0.001**
**I**	7 (0.3%)	7 (0.8%)	0 (0%)	
**II**	134 (6.5%)	84 (9.1%)	50 (4.5%)	
**III**	502 (24.5%)	226 (24.4%)	276 (24.6%)	
**IV**	881 (43.0%)	381 (41.2%)	500 (44.6%)	
**V**	523 (25.5%)	227 (24.5%)	296 (26.4%)	
**FAS** [*n* (%)]				0.112
**Low**	199 (9.7%)	76 (8.2%)	123 (11.0%)	
**Medium**	1133 (55.3%)	519 (56.1%)	614 (54.7%)	
**High**	715 (35.0%)	330 (35.7%)	385 (34.3%)	
**MDS** * (points)	4 (3–5)	4 (3–5)	4 (3–5)	0.071
**Energy intake** (kcal/day)	2180.1 (1634.9–2569.7)	2517.9 (1921.0–2984.3)	1901.6 (1492.4–2244.9)	**<0.001**
**Screen time** (min/day)	256.2 (139.3–330.0)	288.1 (171.4–367.4)	229.9 (126.4–300.0)	**<0.001**

Median values (*p*25–*p*75) expressed. Abbreviations: BMI, body mass index; WC, waist circumference; FMI, fat mass index; FAS, family affluence scale; MDS, Mediterranean diet score. * Mediterranean diet score resulting from the sum of 9 food subgroups compliance. Score ranging from 0–9 points. Significant values (*p* < 0.05) expressed in bold font.

**Table 2:** Multiple linear regression models showing the associations of the Mediterranean diet score (MDS) with screen time.

	Screen Time (min/day)				
	Model I ^a^		Model II ^b^		
	β	*p*	β	*p*	R^2^
**Male**					0.029
MDS (point)	−12.535	**<0.001**	−12.402	**<0.001**	
Energy Intake (kcal/day)	-	-	0.026	**<0.001**	
**Female**					0.016
MDS (point)	−12.402	**<0.001**	−12.402	**<0.001**	
Energy Intake (kcal/day)	-	-	-	-	

^a^ Model I, unadjusted model, studies the association between screen time and MDS. ^b^ Model II presents the variables statistically significant in relation to sedentary time as follows: an initial model was constructed between screen time and MD considering Tanner stage, FAS categories and energy intake as covariates. Furthermore, a step-by-step algorithm was applied to discard non-significant associations. Only statistically significant variables are shown in the present table. Significant values (*p* < 0.05) expressed in bold font.

**Table 3:** Multiple linear regression models of screen time and Mediterranean diet score (MDS) interaction and covariates to predict body mass index, waist circumference and fat mass index displayed by sex.

	Males	*(p-Values)*		Females	*(p-Values)*	
	BMI (kg/m^2^)	WC (cm)	FMI (kg/m^2^)	BMI (kg/m^2^)	WC (cm)	FMI (kg/m^2^)
**Covariates**						
Pubertal Stage						
II *	0.502	0.139	**0.048**	-	-	-
III	0.547	0.209	**0.010**	**0.026**	**0.012**	**0.126**
IV	0.534	0.936	**0.047**	**<0.001**	**<0.001**	**<0.001**
V	0.638	0.866	**0.008**	**<0.001**	**<0.001**	**<0.001**
FAS						
Medium	**0.048**	0.604	**0.045**	**<0.001**	0.138	**<0.001**
High	**0.024**	0.695	**0.019**	**<0.001**	0.058	**<0.001**
Energy Intake (kcal/day)	**<0.001**	**<0.001**	**<0.001**	**<0.001**	**<0.001**	**<0.001**
**Studied Variables**						
MDS (point)	**0.049**	0.260	0.198	**0.043**	0.160	**0.042**
Screen time: MDS	0.062	0.617	0.122	**0.025**	**0.002**	**0.022**

Abbreviations: BMI, body mass index; WC, waist circumference; FMI, fat mass index; FAS, family affluence scale; MDS Mediterranean diet score. * Tanner II *p*-values were not considered to be estimated in the female group, as a statistically small number of females were present in this stage for the current analysis. Significant values (*p* < 0.05) expressed in bold font.

## Data Availability

The data presented in this study are available for further scientific analysis on request from the coordinator of the HELENA study to the following e-mail: lmoreno@unizar.es.

## Supplementary Materials

The following are available online at https://www.mdpi.com/2072-6643/13/2/474/s1, Table S1: Mediterranean diet score (MDS) subgroups displayed in g/day and sex-specific median intake in those HELENA adolescents with dietary data.

## Appendix A

List of researchers HELENA study group.

Co-ordinator: Luis A. Moreno.
Core Group members: Luis A. Moreno, Fréderic Gottrand, Stefaan De Henauw, Marcela González-Gross, Chantal Gilbert.
Steering Committee: Anthony Kafatos (President), Luis A. Moreno, Christian Libersa, Stefaan De Henauw, Sara Castelló, Fréderic Gottrand, Mathilde Kersting, Michael Sjöstrom, Dénes Molnár, Marcela González-Gross, Jean Dallongeville, Chantal Gilbert, Gunnar Hall, Lea Maes, Luca Scalfi.
Project Manager: Pilar Meléndez.

1. Universidad de Zaragoza (Spain)
  Luis A. Moreno, Jose A. Casajús, Jesús Fleta, Gerardo Rodríguez, Concepción Tomás, María I. Mesana, Germán Vicente-Rodríguez, Adoración Villarroya, Carlos M. Gil, Ignacio Ara, Juan Fernández Alvira, Gloria Bueno, Olga Bueno, Juan F. León, Jesús Mª Garagorri, Idoia Labayen, Iris Iglesia, Silvia Bel, Luis A. Gracia Marco, Theodora Mouratidou, Alba Santaliestra-Pasías, Iris Iglesia, Esther González-Gil, Pilar De Miguel-Etayo, Cristina Julián, Mary Miguel-Berges, Isabel Iguacel, Azahara Ruperez and Miguel Seral-Cortes.
2. Consejo Superior de Investigaciones Científicas (Spain)
  Ascensión Marcos, Julia Wärnberg, Esther Nova, Sonia Gómez, Ligia Esperanza Díaz, Javier Romeo, Ana Veses, Belén Zapatera, Tamara Pozo, David Martínez.
3. Université de Lille 2 (France).
  Laurent Beghin, Christian Libersa, Frédéric Gottrand, Catalina Iliescu, Juliana Von Berlepsch.
4. Research Institute of Child Nutrition Dortmund, Rheinische Friedrich-Wilhelms-Universität Bonn (Germany)
  Mathilde Kersting, Wolfgang Sichert-Hellert, Ellen Koeppen.
5. Pécsi Tudományegyetem (University of Pécs) (Hungary) Dénes Molnar, Eva Erhardt, Katalin Csernus, Katalin Török, Szilvia Bokor, Mrs. Angster, Enikö Nagy, Orsolya Kovács, Judit Répasi.
6. University of Crete School of Medicine (Greece)
  Anthony Kafatos, Caroline Codrington, María Plada, Angeliki Papadaki, Katerina Sarri, Anna Viskadourou, Christos Hatzis, Michael Kiriakakis, George Tsibinos, Constantine Vardavas, Manolis Sbokos, Eva Protoyeraki, Maria Fasoulaki.
7. Institut für Ernährungs- und Lebensmittelwissenschaften – Ernährungphysiologie. Rheinische Friedrich Wilhelms Universität (Germany)
  Peter Stehle, Klaus Pietrzik, Marcela González-Gross, Christina Breidenassel, Andre Spinneker, Jasmin Al-Tahan, Miriam Segoviano, Anke Berchtold, Christine Bierschbach, Erika Blatzheim, Adelheid Schuch, Petra Pickert.
8. University of Granada (Spain)
  Manuel J. Castillo, Ángel Gutiérrez, Francisco B Ortega, Jonatan R Ruiz, Enrique G Artero, Vanesa España, David Jiménez-Pavón, Palma Chillón, Cristóbal Sánchez-Muñoz, Magdalena Cuenca.
9. Istituto Nazionalen di Ricerca per gli Alimenti e la Nutrizione (Italy)
  Davide Arcella, Elena Azzini, Emma Barrison, Noemi Bevilacqua, Pasquale Buonocore, Giovina Catasta, Laura Censi, Donatella Ciarapica, Paola D’Acapito, Marika Ferrari, Myriam Galfo, Cinzia Le Donne, Catherine Leclercq, Giuseppe Maiani, Beatrice Mauro, Lorenza Mistura, Antonella Pasquali, Raffaela Piccinelli, Angela Polito, Romana Roccaldo, Raffaella Spada, Stefania Sette, Maria Zaccaria.
10. University of Napoli "Federico II" Dept of Food Science (Italy)
  Luca Scalfi, Paola Vitaglione, Concetta Montagnese.
11. Ghent University (Belgium)
  Ilse De Bourdeaudhuij, Stefaan De Henauw, Tineke De Vriendt, Lea Maes, Christophe Matthys, Carine Vereecken, Mieke de Maeyer, Charlene Ottevaere, Inge Huybrechts.
12. Medical University of Vienna (Austria)
  Kurt Widhalm, Katharina Phillipp, Sabine Dietrich, Birgit Kubelka
  Marion Boriss-Riedl.
13. Harokopio University (Greece)
  Yannis Manios, Eva Grammatikaki, Zoi Bouloubasi, Tina Louisa Cook, Sofia Eleutheriou, Orsalia Consta, George Moschonis, Ioanna Katsaroli, George Kraniou, Stalo Papoutsou, Despoina Keke, Ioanna Petraki, Elena Bellou, Sofia Tanagra, Kostalenia Kallianoti, Dionysia Argyropoulou, Stamatoula Tsikrika, Christos Karaiskos.
14. Institut Pasteur de Lille (France)
  Jean Dallongeville, Aline Meirhaeghe.
15. Karolinska Institutet (Sweden) Michael Sjöstrom, Jonatan R Ruiz, Francisco B. Ortega, María Hagströmer, Anita Hurtig Wennlöf, Lena Hallström, Emma Patterson, Lydia Kwak, Julia Wärnberg, Nico Rizzo.
16. Asociación de Investigación de la Industria Agroalimentaria (Spain) Jackie Sánchez-Molero, Sara Castelló, Elena Picó, Maite Navarro, Blanca Viadel, José Enrique Carreres, Gema Merino, Rosa Sanjuán, María Lorente, María José Sánchez.
17. Campden BRI (United Kingdom)
  Chantal Gilbert, Sarah Thomas, Elaine Allchurch, Peter Burgess.
18. SIK - Institutet foer Livsmedel och Bioteknik (Sweden)
  Gunnar Hall, Annika Astrom, Anna Sverkén, Agneta Broberg.
19. Meurice Recherche & Development asbl (Belgium)
  Annick Masson, Claire Lehoux, Pascal Brabant, Philippe Pate, Laurence Fontaine.
20. Campden & Chorleywood Food Development Institute (Hungary)
  Andras Sebok, Tunde Kuti, Adrienn Hegyi.
21. Productos Aditivos SA (Spain)
  Cristina Maldonado, Ana Llorente.
22. Cárnicas Serrano SL (Spain)
  Emilio García.
23. Cederroth International AB (Sweden)
  Holger von Fircks, Marianne Lilja Hallberg, Maria Messerer.
24. Lantmännen Food R&D (Sweden)
  Mats Larsson, Helena Fredriksson, Viola Adamsson, Ingmar Börjesson.
25. European Food Information Council (Belgium)
  Laura Fernández, Laura Smillie, Josephine Wills.
26. Universidad Politécnica de Madrid (Spain)
  Marcela González-Gross, Raquel Pedrero-Chamizo, Agustín Meléndez, Jara Valtueña, David Jiménez-Pavón, Ulrike Albers, Pedro J. Benito, Juan José Gómez Lorente, David Cañada, Alejandro Urzanqui, Rosa María Torres, Paloma Navarro.
