# Supplementary material for: Mediterranean Diet, Screen-Time-Based Sedentary Behavior and Their Interaction Effect on Adiposity in European Adolescents: The HELENA Study
Source: Nutrients. 2021 Jan 30;13(2):474. doi: 10.3390/nu13020474 (PMC7911943; doi:10.3390/nu13020474)
Supplement: Supplementary file 1 [file nutrients-13-00474-s001.pdf]

Supplementary material

Table S1. Mediterranean Diet Score (MDS) subgroups displayed in g/day and sex-specific median intake in those HELENA adolescents with dietary data.

	Male	Female	
	n=925	n=1122	**p-value**
**Vegetables** (g/day)	77.75<br>(42.78-118.91)	81.85<br>(52.70-122.79)	**0.010**
**Fruits and nuts** (g/day)	103.50<br>(41.69-182.30)	115.02<br>(56.05-187.22)	**0.012**
**Cereal roots** (g/day)	319.24<br>(255.32-397.64)	256.51<br>(205.96-311.57)	**≤0.001**
**Pulses** (g/day)	1.75<br>(0.76-3.61)	1.44<br>(0.67-3.25)	**0.029**
**Fish** (g/day)	12.35<br>(3.87-29.23)	13.18<br>(4.91-26.34)	0.971
**Dairy products** (g/day)	214.72<br>(98.22-364.52)	154.97<br>(72.86-260.45)	**≤0.001**
**Meat** (g/day)	156.63<br>(106.96-204.73)	123.56<br>(82.21-169.14)	**≤0.001**
**Alcohol** (g/day)	0.12<br>(0.03-0.45)	0.23<br>(0.02-0.42)	**≤0.001**
**FU/FS ratio**	0.86<br>(0.77-0.98)	0.87<br>(0.78-1.00)	0.142
**MDS**	4<br>(0-8)	4<br>(0-8)	0.071
**High MD adherence** (n, %)	382<br>(41.29%)	508<br>(45.27%)	0.105

FU/FS ratio: unsaturated to saturated fatty acids ratio**.** Median values (p25-p75) displayed.
